# Association of weight change with cerebrospinal fluid biomarkers and amyloid positron emission tomography in preclinical Alzheimer’s disease

**DOI:** 10.1186/s13195-021-00781-z

**Published:** 2021-02-17

**Authors:** Oriol Grau-Rivera, Irene Navalpotro-Gomez, Gonzalo Sánchez-Benavides, Marc Suárez-Calvet, Marta Milà-Alomà, Eider M. Arenaza-Urquijo, Gemma Salvadó, Aleix Sala-Vila, Mahnaz Shekari, José Maria González-de-Echávarri, Carolina Minguillón, Aida Niñerola-Baizán, Andrés Perissinotti, Maryline Simon, Gwendlyn Kollmorgen, Henrik Zetterberg, Kaj Blennow, Juan Domingo Gispert, José Luis Molinuevo, for the ALFA Study, Annabella Beteta, Raffaele Cacciaglia, Alba Cañas, Carme Deulofeu, Irene Cumplido, Ruth Dominguez, Maria Emilio, Carles Falcon, Sherezade Fuentes, Laura Hernandez, Gema Huesa, Jordi Huguet, Karine Fauria, Paula Marne, Tania Menchón, Grégory Operto, Albina Polo, Sandra Pradas, Anna Soteras, Marc Vilanova, Natàlia Vilor-Tejedor

**Affiliations:** 1 Barcelonaβeta Brain Research Center (BBRC), Pasqual Maragall Foundation, Barcelona, Spain; 2 Servei de Neurologia, Hospital del Mar, Barcelona, Spain; 3 IMIM (Hospital del Mar Medical Research Institute), Barcelona, Spain; 4 Centro de Investigación Biomédica en Red de Fragilidad y Envejecimiento Saludable (CIBERFES), Madrid, Spain; 5 Universitat Pompeu Fabra, Barcelona, Spain; 6 Servei de Medicina Nuclear, Hospital Clínic, Barcelona, Spain; 7 Centro de Investigación Biomédica en Red de Bioingeniería, Biomateriales y Nanomedicina (CIBER-BBN), Madrid, Spain; 8 Roche Diagnostics International Ltd, Rotkreuz, Switzerland; 9 Roche Diagnostics GmbH, Penzberg, Germany; 10 Clinical Neurochemistry Laboratory, Sahlgrenska University Hospital, Mölndal, Sweden; 11 UK Dementia Research Institute at UCL, London, UK; 12 Department of Psychiatry and Neurochemistry, Institute of Neuroscience and Physiology, University of Gothenburg, Mölndal, Sweden; 13 Department of Neurodegenerative Disease, UCL Queen Square Institute of Neurology, London, UK; 14 Current affiliation: H. Lundbeck A/S, Copenhagen, Denmark

**Keywords:** Alzheimer’s disease, Preclinical, Cognitively unimpaired, Weight loss, Biomarkers, Risk factors

## Abstract

**Background:**

Recognizing clinical manifestations heralding the development of Alzheimer’s disease (AD)-related cognitive impairment could improve the identification of individuals at higher risk of AD who may benefit from potential prevention strategies targeting preclinical population. We aim to characterize the association of body weight change with cognitive changes and AD biomarkers in cognitively unimpaired middle-aged adults.

**Methods:**

This prospective cohort study included data from cognitively unimpaired adults from the ALFA study (*n* = 2743), a research platform focused on preclinical AD. Cognitive and anthropometric data were collected at baseline between April 2013 and November 2014. Between October 2016 and February 2020, 450 participants were visited in the context of the nested ALFA+ study and underwent cerebrospinal fluid (CSF) extraction and acquisition of positron emission tomography images with [^18^F]flutemetamol (FTM-PET). From these, 408 (90.1%) were included in the present study. We used data from two visits (average interval 4.1 years) to compute rates of change in weight and cognitive performance. We tested associations between these variables and between weight change and categorical and continuous measures of CSF and neuroimaging AD biomarkers obtained at follow-up. We classified participants with CSF data according to the AT (amyloid, tau) system and assessed between-group differences in weight change.

**Results:**

Weight loss predicted a higher likelihood of positive FTM-PET visual read (OR 1.27, 95% CI 1.00–1.61, *p* = 0.049), abnormal CSF p-tau levels (OR 1.50, 95% CI 1.19–1.89, *p* = 0.001), and an A+T+ profile (OR 1.64, 95% CI 1.25–2.20, *p* = 0.001) and was greater among participants with an A+T+ profile (*p* < 0.01) at follow-up. Weight change was positively associated with CSF Aβ42/40 ratio (β = 0.099, *p* = 0.032) and negatively associated with CSF p-tau (β = − 0.141, *p* = 0.005), t-tau (β = − 0.147 *p* = 0.004) and neurogranin levels (β = − 0.158, *p* = 0.002). In stratified analyses, weight loss was significantly associated with higher t-tau, p-tau, neurofilament light, and neurogranin, as well as faster cognitive decline in A+ participants only.

**Conclusions:**

Weight loss predicts AD CSF and PET biomarker results and may occur downstream to amyloid-β accumulation in preclinical AD, paralleling cognitive decline. Accordingly, it should be considered as an indicator of increased risk of AD-related cognitive impairment.

**Trial registration:**

NCT01835717, NCT02485730, NCT02685969.

## Background

Decline in body weight is common among patients with Alzheimer’s disease (AD) and has been associated with disease progression and severity [1–5]. Evidence from long-term longitudinal epidemiological studies suggests that weight loss precedes the onset of dementia up to a decade or more and accelerates by the time of diagnosis [2, 6–11]. Furthermore, weight decline from midlife to late-life has been linked with increased risk of incident mild cognitive impairment [12], and there is neuropathological evidence that links lower body mass index (BMI) with a higher level of AD pathology, but not with other common causes of dementia, such as vascular pathology and Lewy body pathology [13]. Regarding mechanisms underlying these associations, it has been suggested that hypothalamic dysfunction and olfactory function impairment due to AD-related neurodegeneration (or in the context of other neurodegenerative conditions) may cause weight decline [14]. According to this point of view, rather than a cause of AD, weight loss would be a clinical manifestation that may appear before the onset of cognitive impairment. In addition, cognitive, behavioral and mood changes arising throughout the course of the disease may also contribute to weight loss.

During the last years, studies incorporating AD biomarkers have shown that higher baseline levels of amyloid-β (Aβ) deposition, as measured by positron emission tomography (PET), and higher cerebrospinal fluid (CSF) levels of tau/Aβ42 ratio are associated with greater prospective body weight decline [15, 16], and a recent study has reported a negative correlation between BMI change and amyloid-PET burden [17]. Other studies have found a negative correlation between BMI and Aβ and tau deposition in the brain of cognitively unimpaired elders [18–21]. While these studies have focused on associations with continuous measures of AD biomarkers, few studies have evaluated whether weight loss predicts AD biomarker positivity using categorical outcomes, such as amyloid-PET visual read results. Determining the extent to which weight change predicts AD biomarker results may be of interest to evaluate weight loss as a potential predictor of cognitive impairment due to AD. Furthermore, analyzing weight change differences among cognitively unimpaired adults with different biomarker profiles may help to clarify when in the preclinical *continuum* does weight loss occur, which is currently not clear. Finally, we are not aware of previous studies having evaluated associations between weight change and biomarkers of axonal damage and synaptic dysfunction, such as neurofilament light (NfL) and neurogranin, which are believed to occur downstream to amyloid and tau deposition [22]. Exploring these associations may help to characterize biological changes associated with weight loss in preclinical AD.

Consequently, our main objective was to investigate to which extent preceding weight loss can predict amyloid positron emission tomography (PET) visual read and cerebrospinal fluid (CSF) AD biomarkers results in a cohort of cognitively unimpaired middle-aged adults at increased risk for AD. Secondary objectives aimed to (i) analyze differences in weight change across individuals with different CSF biomarkers profiles, (ii) characterize the association of weight change with continuous measurements of CSF and PET biomarkers and longitudinal cognitive performance changes, and (iii) explore whether these associations are modified by amyloid-β (Aβ) status, AD family history and age.

## Methods

### Participants

We included participants from the ALFA study (for ALzheimer and FAmilies) who were subsequently enrolled in the nested ALFA+ cohort study. Details of both studies design have been published elsewhere [23]. The ALFA study comprises 2743 cognitively unimpaired adults aged between 45 and 75 years, most of them offspring of AD patients, who underwent baseline anthropometric measurements, blood sampling, *APOE* genotyping, and neuropsychological testing between April 2013 and November 2014. From these, 450 participants were enrolled in the ALFA+ study (*n* = 450). The ALFA+ study inclusion criteria involves cognitively healthy adults (CDR = 0, MMSE≥27, semantic fluency≥12 animals/minute) without any significant systemic illness, unstable medical condition or major psychiatric disorder. A positive parental family history of sporadic AD (self-reported parental history of AD supported either by a documented clinical diagnosis or a retrospective clinical diagnosis consistent with AD dementia) has been documented in 95% of participants (68% of them with parental AD onset before 75 years old). For the present study, we categorized parental AD family history as negative (FH-), positive with onset ≥75 years-old (FH ≥ 75) or positive with onset < 75 years-old (FH < 75). ALFA+ participants undergo blood and CSF extraction, as well as a detailed clinical, neuropsychological, and neuroimaging characterization every 3 years. Data and sample collection from the first ALFA+ study visit was performed between October 2016 and January 2020. For the present study, we included participants with available weight measurements on two consecutive visits (mean interval 4.1 years, SD 0.9, range 2.2–6 years): the ALFA study baseline visit (hereafter “baseline”) and the first ALFA+ visit (hereafter “follow-up”), as well as available data on cognitive performance (both from baseline and follow-up visits) and/or AD biomarkers (only available at follow-up visit). We excluded participants with (i) insufficient data (*n* = 33), (ii) extreme weight change values, as defined in the “Statistical analyses” section (*n* = 8; absolute weight change of 20–48 Kg), and (iii) evidence of non-AD causes of cognitive impairment (one participant who subsequently developed frontotemporal dementia), rendering a final sample of 408 participants.

### Clinical assessments

We computed the following annualized rate of weight change, where negative values express the percentage of weight loss per year:

$$ \mathrm{Weight}\ \mathrm{change}=\frac{100\times \left(\mathrm{Follow}-\mathrm{up}\ \mathrm{weight}-\mathrm{Baseline}\ \mathrm{weight}\right)/\mathrm{Baseline}\ \mathrm{weight}}{\mathrm{Years}\ \mathrm{between}\ \mathrm{visits}} $$

We used a composite similar to the Preclinical Alzheimer’s Cognitive Composite score (PACC-like) to evaluate cognitive performance, based on longitudinally available measures of episodic verbal memory, timed executive function and semantic processing. The PACC-like composite includes the total immediate paired recall (0–32) and total delayed free recall (0–32) scores from the Memory Binding Test, the coding subtest from the Wechsler Adult Intelligence Scale-IV (0–135), and a semantic fluency test (number of animals per minute). We decided to include a semantic fluency test instead of the MMSE, as it provides more accurate information regarding early AD-related cognitive decline, based on recent literature [24–26]. To calculate the PACC-like longitudinal change, we standardized the four scores by dividing the difference between subject score and baseline mean by the baseline standard deviation, summed-up these standardized scores, and divided the resulting values by the number of years between visits, as described elsewhere [27]. This can be summarized as follows, where *i* corresponds to each test used to calculate the composite:

$$ \mathrm{PACC}-\mathrm{like}\ \mathrm{change}=\frac{\sum \left[\left({\mathrm{Baseline}\ \mathrm{score}}_i-\mathrm{Follow}-\mathrm{up}\ {\mathrm{score}}_i\right)/\mathrm{Baseline}\ \mathrm{score}\ {\mathrm{standard}\ \mathrm{deviation}}_i\right]}{\mathrm{Years}\ \mathrm{between}\ \mathrm{visits}} $$

In addition, variables related to mood, physical activity and dietary habits were collected at the follow-up visit. Mood was assessed with the Spanish version of the Hospital Anxiety and Depression Scale (HADS) [28]. HADS anxiety and depression subscales were summed to obtain a total HADS score. Physical exercise level was measured using the Spanish short version of the Minnesota Leisure Time Physical Activity Questionnaire [29]. The questionnaire collects the following information: (i) type of activity, (ii) frequency of activity, (iii) duration of activity, and (iv) intensity of activity. Using these variables, energy expenditure can be calculated following the formula: Energy expenditure (MET) = Frequency (days) × duration (minutes) × intensity (Kcal), with 1 MET-min roughly equating to 1 Kcal. Dietary habits were assessed using the Mediterranean Diet Adherence Screener (MEDAS) score, a validated questionnaire that includes 14 questions on food consumption frequency and food intake habits considered characteristic of the Spanish Mediterranean diet [30].

### CSF biomarker measurement and cutoff definitions

CSF biomarker data were available for 387 participants. Samples were processed as described elsewhere [22]. We measured CSF levels of Aβ42, Aβ40, p-tau, t-tau, NfL, and neurogranin using the Roche NeuroToolKit and Elecsys® immunoassays. We used in-house published CSF cutoffs to define biomarker profiles [22]. We defined Aβ positivity (A+) as CSF Aβ42/40 < 0.071 and tau positivity (T+) as CSF p-tau > 24 pg/mL and further classified participants in four AT biomarker profiles: A-T-, A+T-, A-T+, and A+T+. We used CSF instead of PET data to define Aβ status for the AT classification in our main analyses for comparability with other studies in the ALFA cohort [22] and because there is evidence that CSF analysis may be more sensitive than PET to detect early Aβ deposition, which is relevant for a analyses involving preclinical population [31]. However, for completeness, we also report additional analyses using PET data to define Aβ status for the AT classification. We used this simplified classification instead of the ATN proposed by the NIA-AA Research Framework [32] because the latter resulted in a very low number of individuals in most of the biomarker profile categories, which hindered between-group statistical comparisons.

### [^18^F] flutemetamol PET images acquisition and processing

[^18^F] flutemetamol PET (FTM-PET) images were acquired in 352 participants and preprocessed as described previously [33]. Tracer uptake was quantified in Centiloid units using SPM12 following a validated Centiloid pipeline [34]. Images were visually rated as Aβ positive or negative by an experienced nuclear medicine physician. Two reads were considered misleading and excluded from analyses.

### Statistical analyses

Data were initially screened to identify influential outliers and departures from normality. We defined extreme values using Tukey’s method as those lower than 3 times the interquartile range below the first quartile or higher than 3 times the interquartile range above the third quartile. To avoid potential measurement errors or outlier effects, besides excluding participants with extreme weight change values (*n* = 8), we omitted from regression analyses extreme values in PACC-like change (*n* = 6), CSF p-tau (*n* = 4), t-tau (*n* = 2), neurogranin (*n* = 2), and NfL (*n* = 3). We applied a logarithmic (base 10) transformation on CSF p-tau, t-tau, NfL and neurogranin measures in order to meet the normality assumption.

We used binary and multinomial logistic regression, as appropriate, to evaluate the extent to which weight loss predicted having a positive visual read of FTM-PET, abnormal CSF p-tau levels, or a pathological AT profile. We chose FTM-PET instead of CSF Aβ42/40 as the main Aβ outcome for this analysis because visual read of amyloid PET has been extensively used in clinical trials and is being increasingly used in the clinical routine, and because the CSF Aβ42/40 cutoff used in the present study is remarkably higher than the one commonly used for diagnostic purposes, because it was derived for research purposes to maximize the early detection Aβ pathology changes [22]. However, for completeness, we also show analyses using CSF Aβ42/40 dichotomic result as an outcome. We used ANCOVA to evaluate differences in the annualized rate of weight change between AT categories followed by post hoc pairwise comparisons using Bonferroni’s method to adjust by multiple comparisons. We performed linear regression models to assess the association of the annualized rate of weight change as the predictor of PACC-like change, as well as CSF biomarker levels and Centiloids at follow-up. We further added an “Aβ status x weight change,” “AD parental family history x weight change,” and “age x weight change” interaction terms to these models and performed stratified analyses by Aβ status using the CSF Aβ42/40 < 0.071 cutoff. ANCOVA and regression analyses were adjusted by age, sex, *APOE*-ε4 status (number of ε4 alleles) and baseline BMI. Regression models involving PACC-like change were additionally adjusted by years of education. To exclude the effect of other potential confounders [35–37], we repeated the ANCOVA and main effect regression analyses additionally adjusting for variables related to cardiovascular health (systolic blood pressure, cholesterol, triglycerides, glycated hemoglobin, physical exercise and dietary habits), as well as HADS total score and AD family history. A 2-tailed *p* < 0.05 was considered significant. We applied a false discovery rate (FDR) correction for multiple comparisons using the Benjamini-Hochberg procedure to our primary objective analyses (associations of annualized rate of weight change with FTM-PET visual read, CSF p-tau dichotomic result and AT profile) [38]. The remaining analyses were not corrected for multiple comparisons and should therefore be considered as exploratory.

Statistical analyses were conducted with Stata 15 software (StataCorp. 2017. Stata Statistical Software: Release 15. College Station, TX: StataCorp LLC) and R statistical software (R Core Team 2014. R: A Language and Environment for Statistical Computing, version 3.2.5. Available at: http://www.r- project.org).

### Standard protocol approvals, registrations, and patient consents

The ALFA study and ALFA+ cohort study protocols have been approved by the Independent Ethics Committee Parc de Salut Mar Barcelona and registered at ClinicalTrials.gov (Identifiers: NCT01835717 and NCT02485730, respectively). All participants signed a written informed consent before their inclusion.

## Results

### Sample description

We included 408 participants in the study, with 77% of them being younger than 65 years. Sample characteristics are described in Table 1.

**Table 1:** Demographic, clinical, genetic, and biomarker data of the entire group

*Characteristic*	No. (%) with available data	*Mean (SD)/no. (%)*
Age at follow-up, years	408 (100)	61.2 (4.7)
Female sex	408 (100)	246 (60.3)
Education, years	408 (100)	13.5 (3.6)
*APOE-*ɛ4 non-carriers	408 (100)	182 (44.6)
*APOE-*ɛ4 heterozygous		190 (46.6)
*APOE-*ɛ4 homozygous		36 (8.8)
Negative AD family history (%)	408 (100)	21 (5.1)
Positive AD family history, onset ≥ 75 years (%)		124 (30.4)
Positive AD family history, onset < 75 years (%)		263 (64.5
CSF Aβ42/40 ratio	387 (95)	0.075 (0.02)
CSF pTau, pg/mL	387 (95)	16.2 (7.6)
CSF tTau, pg/mL	387 (95)	197.3 (73.3)
CSF NfL, pg/mL	387 (95)	82.7 (30.5)
CSF neurogranin, pg/mL	387 (95)	798.7 (331.6)
FTM-PET uptake, centiloids	352 (86)	2.9 (17.0)
FTM-PET positive visual reads	350 (86)	45 (12.9)
PACC-like change/year	406 (99.5)	0.108 (0.5)
BMI at baseline, Kg/m^2^	408 (100)	26.8 (4.0)
% weight change/year	408 (100)	0.049 (1.5)
Systolic blood pressure, mmHg	406 (99.5)	127.3 (14.3)
Total cholesterol, mg/dL	408 (100)	203.5 (30.5)
Triglycerides, mg/dL	408 (100)	99.4 (58.8)
HbA1c, %	402 (98.5)	5.5 (0.4)
HADS total score	407 (99.8)	7.0 (5.1)
Physical exercise, MET per week	408 (100)	3768.0 (2806.8)
**MEDAS score**	**395 (96.8)**	**9.3 (2.0)**

*SD* standard, *FTM-PET* [18F] flutemetamol positron emission tomography, *BMI* body mass index, *HbA1c* glycated hemoglobin, *HADS* Hospital Anxiety and Depression scale, *MET* metabolic equivalent of task, *MEDAS* Mediterranean Diet Adherence Screener

### Prediction of biomarker status by weight loss

**Fig. 1:**
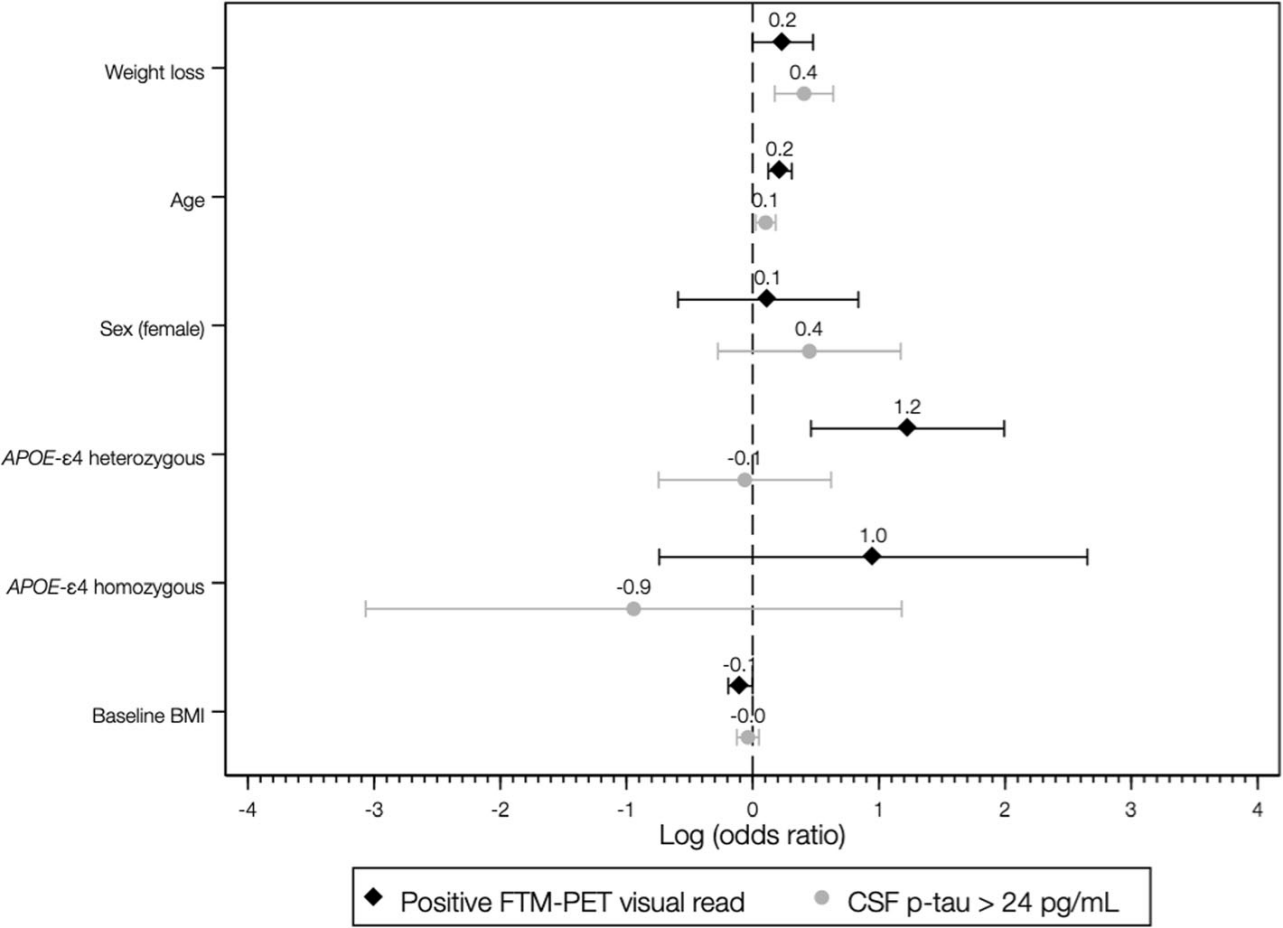
Odds ratios (in logarithmic scale) and 95% confidence intervals for the association between weight loss (% weight loss during follow-up) and core-AD biomarker results. Weight loss refers to reversed values of the annualized weight change rate (i.e., positive values of weight change expressed weight loss)

**Fig. 2:**
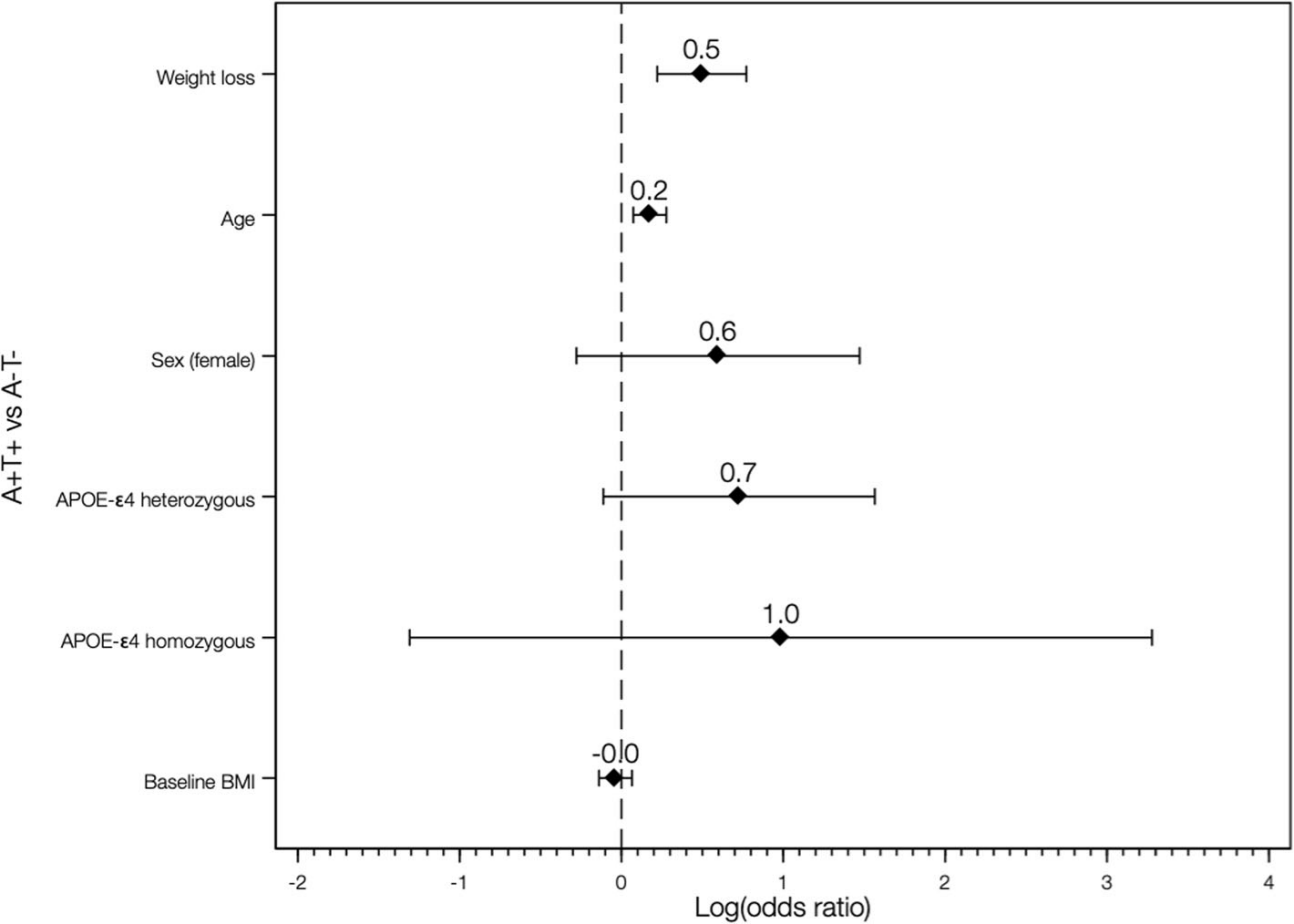
Odds ratios (in logarithmic scale) and 95% confidence intervals for the association between weight loss (% weight loss during follow-up) and being A+T+ compared with A-T-. Weight loss refers to reversed values of the annualized weight change rate (i.e., positive values of weight change expressed weight loss)

Weight loss predicted a higher likelihood of having a positive FTM-PET visual read [odds ratio (OR) 1.27, 95% confidence interval (CI) 1.00–1.61, *p* = 0.049], displaying abnormal CSF p-tau levels (OR 1.50, 95% CI 1.19–1.89, *p* = 0.001), and having an A+T+ (as compared to an A-T-) CSF biomarker profile (OR 1.64, 95% CI 1.25–2.20, *p* = 0.001) (Figs. 1 and 2). In contrast, the association between weight loss and increased likelihood of abnormal CSF Aβ42/40 levels did not reach statistical significance (OR 1.13, 95% CI 0.97–1.33, *p* = 0.118).

### Weight differences across AT profiles

**Fig. 3:**
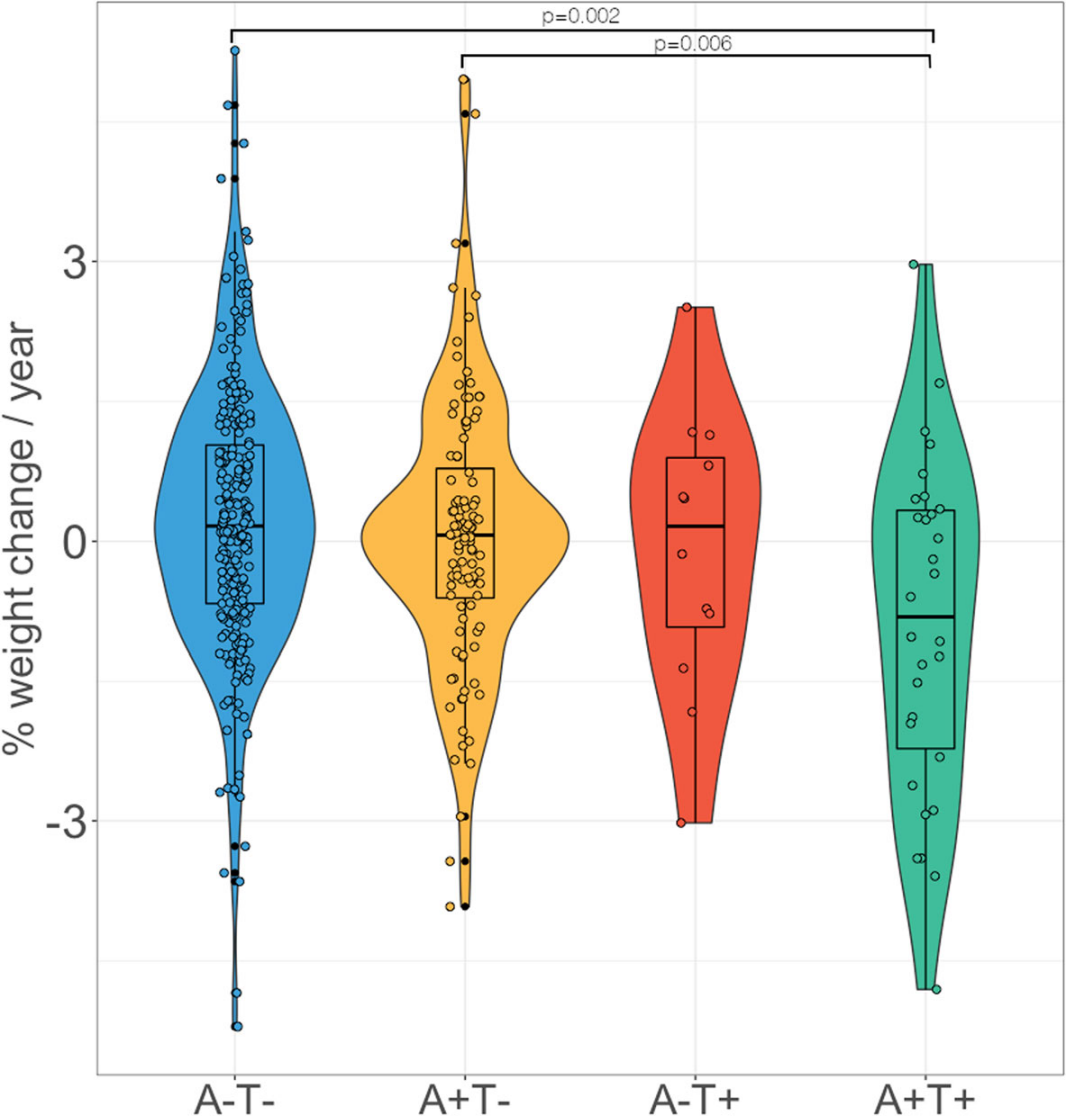
Differences in the annualized rate of weight change across AT groups. Brackets and numbers above them indicate between-group statically significant differences and Bonferroni-adjusted *P* values

We found statistically significant differences in the annualized rate of weight change between AT profiles (*F* = 4.85, *p* = 0.002) with significant weight loss being observed only in the A+T+ group (mean − 0.91, SD 1.8) compared with the A+T- (mean 0.06, SD 1.4, *p* = 0.005) and A-T- (mean 0.22, SD 1.5, *p* = 0.001) profiles (Fig. 3). We did not observe significant differences in the annualized rate of change between A-T+ participants (mean − 0.11, SD 1.5) compared with other AT profiles (A-T+ vs A-T- *p* = 0.944, A-T+ vs A+T- *p* = 0.974, A-T+ vs A+T+ *p* = 0.668).

### Association of weight change with cognitive change and cross-sectional biomarker measures

The annualized rate of weight change was not associated with PACC-like change in the whole group (standardized β = 0.003, *p* = 0.948). In contrast, weight change was positively associated with CSF Aβ42/40 ratio (standardized β = 0.099, *p* = 0.032) and negatively associated with CSF p-tau (standardized β = − 0.141, *p* = 0.005), t-tau (standardized β = − 0.147, *p* = 0.004), and neurogranin levels (standardized β = − 0.158, *p* = 0.002), but not with NfL levels (standardized β = − 0.068, *p* = 0.139). We also found a trend for a positive association between weight change and Centiloids (standardized β = − 0.098, *p* = 0.060). In other words, participants who had experienced greater weight loss tend to display more abnormal levels of core AD biomarkers and synaptic loss biomarkers in the follow-up-visit.

### Interactions of weight change with Aβ status, AD parental family history and age

**Fig. 4:**
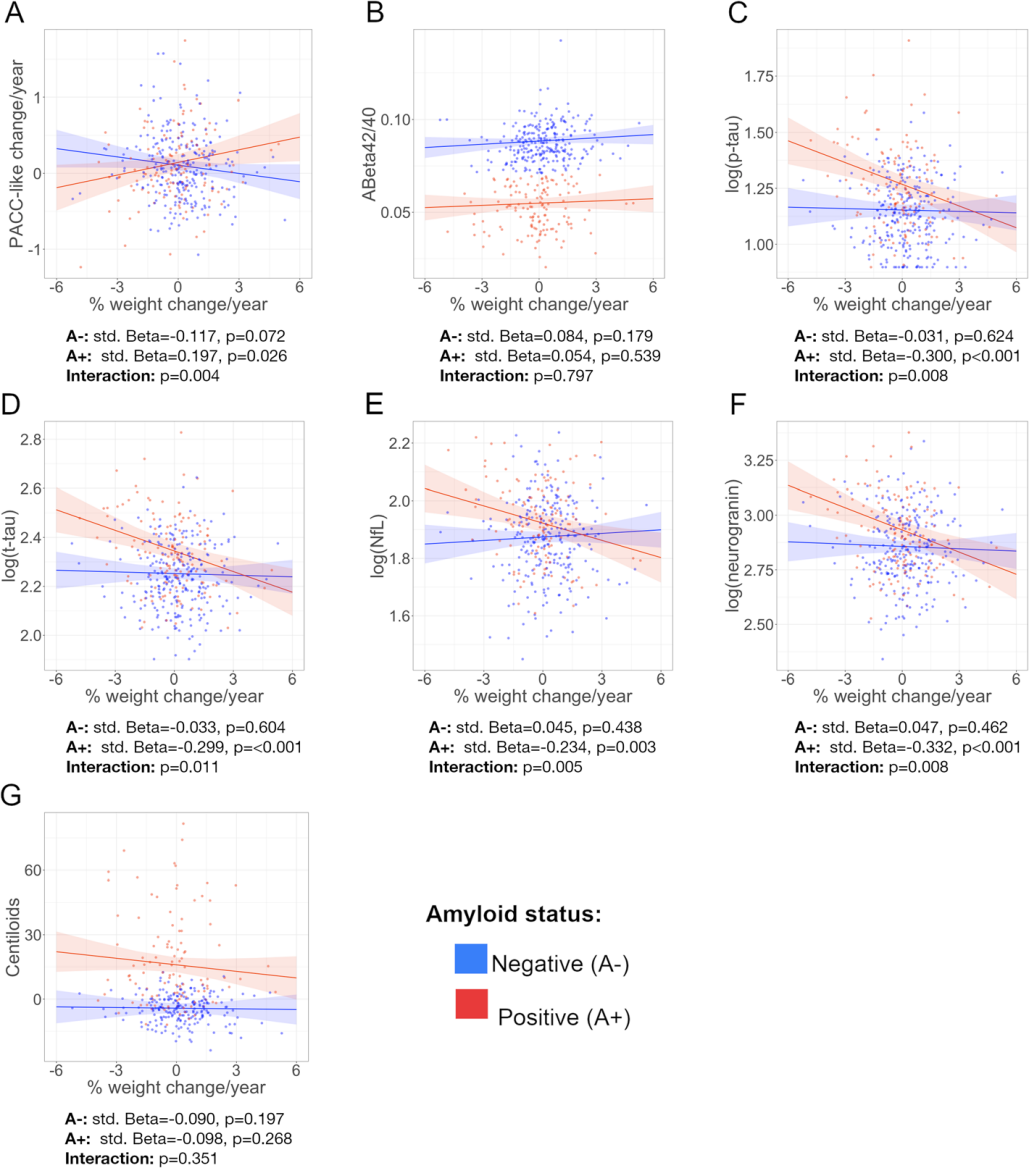
Interaction between amyloid status and the annualized rate of weight change on cognitive performance change, CSF biomarkers and Centiloids. std. Beta: standardized beta coefficients

We found significant interactions between weight change and Aβ status on PACC-like change (standardized β = 0.193, *p* = 0.004) and CSF p-tau (standardized β = − 0.162, *p* = 0.008), t-tau (standardized β = − 0.156, *p* = 0.011), NfL (standardized β = − 0.160, *p* = 0.005), and neurogranin (standardized β = − 0.166, *p* = 0.008) (Fig. 4a, c–f), but not on CSF or PET Aβ measures (*p* > 0.05 for both interaction terms) (Fig. 4b, g). After stratifying by Aβ status, we found that weight loss was significantly associated with faster cognitive decline in the A+ group (standardized β = 0.197, *p* = 0.026), while an opposite trend was observed in the A- group (standardized β = − 0.117, *p* = 0.072) (Fig. 4a). Stratified analyses also revealed significant negative associations between weight change and CSF p-tau (standardized β = − 0.300, *p* < 0.001), t-tau (standardized β = − 0.299, *p* < 0.001), NfL (standardized β = − 0.234, *p* = 0.003), and neurogranin (standardized β = − 0.332, *p* < 0.001), in the A+ group only (Fig. 4c–f). We found a significant interaction between AD parental family history and weight change on CSF NfL levels (*p* < 0.05); however, we did not observe any significant association between weight change and CSF NfL levels after stratifying by AD parental family history (FH-: standardized β = 0.328, *p* = 0.217; FH ≥ 75: standardized β = − 0.127, *p* = 0.130; FH < 75: standardized β = − 0.068, *p* = 0.225). We did not find any other significant interactions between weight change and AD family history or age on PACC-like change, CSF biomarkers, or FTM-PET uptake levels (*p* > 0.05 for all interaction terms).

### Additional analyses

We performed additional analyses using PET instead of CSF data to define Aβ status for the AT classification and found significant differences among AT groups on weight change (*F* = 3.84, *p* = 0.01). The estimated effect size was similar using either PET (partial η2 = 0.037) or CSF data (partial η2 = 0.035). In pairwise analyses, we only found statistically significantly greater weight loss in A+T+ compared with A-T- participants (*p* = 0.049). Differences in the results when using PET instead of CSF data may be explained by a lower statistical power due to a lower number Aβ positive individuals when using PET as compared to CSF data, since the effect size was similar with both methods.

We also ran additional analyses to study the association between weight change and variables related with cardiovascular risk (systolic blood pressure, cholesterol, triglycerides, HbA1c, dietary habits and physical exercise), mood (HADS total score), and AD family history and to exclude the effect of these potential confounders in our main analyses. In age- and sex-adjusted models, we found a statistically significant positive association between weight change and triglyceride levels (standardized β = 0.166, *p* = 0.001) and a trend for a positive association with total cholesterol (standardized β = 0.096, *p* = 0.062), meaning that participants who had experienced greater weight loss tended to have lower triglyceride and cholesterol levels at follow-up. We did not find significant associations between weight change and HbA1c levels (standardized β = 0.082, *p* = 0.108), systolic blood pressure (standardized β = 0.0001, *p* = 0.998), physical exercise (standardized β = − 0.064, *p* = 0.201), HADS total score (standardized β = 0.041, *p* = 0.424), dietary habits (MEDAS score) (standardized β = − 0.026, *p* = 0.615), or AD parental family history (FH ≥ 75 vs FH-: standardized β = − 0.134, *p* = 0.218; FH < 75 vs FH-: − 0.085, *p* = 0.433). After adjusting by all these potential confounders, associations between the annualized weight rate of weight change and most of the categorical biomarker outcomes remained statistically significant: OR of 1.33 for positive FTM-PET visual read (95% CI 1.04–1.73, *p* = 0.025), OR of 1.13 for abnormal CSF Aβ42/40 ratio levels (95% CI 0.95–1.34, *p* = 0.171), OR of 1.48 for abnormal CSF p-tau levels (95% CI 1.16–1.88, *p* = 0.001), and OR of 1.61 for being A+T+ vs A-T- biomarker profile (95% CI 1.20–2.15, *p* = 0.001). Weight change differences between AT groups remained statistically significant (*F* = 4.89, *p* = 0.002), as well as a trend for a positive association with CSF Aβ42/40 (standardized β = 0.089, *p* = 0.067), and negative associations with CSF p-tau (standardized β = − 0.143, *p* = 0.008), t-tau (standardized β = − 0.146, *p* = 0.007), and neurogranin (standardized β = − 0.141, *p* = 0.008) levels in the whole sample. In addition, the negative association with Centiloids was significant (β = − 1.127, *p* = 0.021). Associations of weight change with PACC-like change and CSF NfL levels remained non-significant (standardized β = 0.002, *p* = 0.972 and standardized β = − 0.060, *p* = 0.222, respectively).

## Discussion

In the present study, we sought to characterize weight change and investigate its association with AD biomarkers in a cohort of cognitively unimpaired adults at increased risk for AD. While prior studies have analyzed how baseline AD biomarkers predict longitudinal weight change, we evaluated whether preceding weight change predicted biomarker status at a given time-point. We found that preceding weight loss was associated with a higher likelihood of displaying abnormal results of AD core biomarkers. Moreover, participants with an A+T+ biomarker profile had experienced greater weight loss than those with A+T- and A-T- profiles, and weight loss was associated with higher burden of AD pathology, as measured with continuous measures of CSF and PET biomarkers. In participants who were in the Alzheimer’s *continuum*, as described by Jack et al in the NIA-AA research framework [32] (i.e., those with at least abnormal CSF Aβ results), weight loss was associated with higher levels of CSF tau-related, axonal damage and synaptic dysfunction biomarkers, as well as faster cognitive decline. Overall, our findings show that weight loss is a clinical feature of preclinical AD that probably occurs downstream to Aβ deposition, which may indicate increased risk of developing AD-related cognitive impairment.

Our results showed a robust association between weight loss and the presence of altered AD biomarkers, with each annual percentual point of decrease in body weight being associated with a higher likelihood of having a positive FTM-PET visual read (OR 1.27), abnormal CSF p-tau levels (OR 1.50), and being A+T+ vs A-T- profile (OR 1.64). Weight loss was a better predictor than age for either having a positive FTM-PET visual read, abnormal CSF p-tau levels, or being A+T+ (OR 1.1-1-2) and represented approximately half of the OR associated with being an *APOE-*ε4 allele carrier for FTM-PET positivity (3.4 for heterozygous and 2.6 for homozygous) or having an A+T+ vs A-T- biomarker profile (2.1 for heterozygous and 2.7 for homozygous). Notably, this amount of risk increase also approached 50% of that reported for individuals carrying one copy of the *APOE-*ε4 allele for developing AD during lifetime (*ε2/ε4*, OR 2.6; *ε3/ε4*, OR 3.2) [39]. However, our data also reflect that weight loss occurs at a relatively slow pace, even in those individuals in a more advanced stage of the Alzheimer’s *continuum* (~ 1% loss/year in A+T+ group). This suggests that weight loss alone is not a useful predictor of AD biomarkers results, as it may take several years in order to reach a clinically meaningful amount of weight loss in the preclinical AD population. However, it is unknown whether adding longitudinal weight measurements may improve the accuracy of previously validated tools that combine cardiovascular and other risk factors to predict long-term dementia risk [40, 41]. This hypothesis deserves further study, since measuring body weight is easy and affordable, and, combined with other risk factors, may provide a useful information to the general practitioner to assess dementia risk in patients with cognitive complaints. In line with this, we hypothesize that among patients with subjective cognitive decline (SCD), which has been associated with increased Aβ burden and dementia risk [42, 43], weight loss would increase the likelihood of AD pathology as the underlying cause. Since this specific question was beyond the scope of the present study, the clinical relevance of weight loss among SCD population should be further investigated.

Epidemiological evidence suggests that weight loss precedes the onset of cognitive impairment in patients with dementia. However, the interval of time between weight decline and dementia onset remains unclear, with data from different studies ranging from 6 to 20 years [7, 9, 10]. In addition, a study comparing BMI in preclinical individuals with autosomal dominant AD and non-carrier healthy adults showed that preclinical mutation carriers display significantly lower BMI approximately 11 years before expected symptoms onset, although BMI trajectories may start to diverge up to one decade before [44]. As far as we are concerned, previous studies on the association between weight change and AD biomarkers have not addressed whether weight loss occurs at a particular stage of preclinical AD [15, 16, 18]. We approached this question by comparing weight change between groups with different biomarker profiles and performing interaction analyses with Aβ status, which provides us with an approximate picture of when does weight loss occur in the Alzheimer’s *continuum*. Our results show that weight loss mainly occurs in individuals with abnormal CSF Aβ and p-tau levels (A+T+). This is also supported by interaction and stratified analyses showing that associations with CSF p-tau, t-tau, neurogranin, and NfL were only significant in A+ individuals, which suggests that weight loss in preclinical AD may occur mostly downstream to Aβ accumulation.

Regarding potential mechanisms underlying the observed associations, it has been suggested that hypothalamic dysfunction may be an important driver of weight loss in preclinical AD, since Aβ and tau deposits have been described in AD in this nucleus, which plays a key role in energy homeostasis [14]. Olfactory impairment, which has been identified as an early sign of AD, may also contribute to weight decline [45].

Conversely, while cognitive impairment and mood disorders are likely contributors to weight loss in symptomatic AD [46], it is unlikely that these specific variables drove the observed associations because the current study was conducted in a sample of cognitively unimpaired adults and adjustment for anxiety and depression did not modify the findings.

We also explored whether weight change was associated with longitudinal cognitive changes, as previous studies have reported that weight loss is associated with faster cognitive decline in cognitively unimpaired elders [15, 47]. We did not find significant associations between longitudinal weight and cognitive changes in the whole sample. However, stratified analyses showed that greater weight loss was associated with faster cognitive decline specifically in A+ participants. This indicates that weight loss may be a marker of incipient AD-related cognitive decline, which is particularly interesting in the context of preclinical AD, where, by definition, cognitive decline is below the clinical threshold for cognitive impairment diagnosis, and therefore detecting and tracking cognitive changes may be challenging [48, 49]. We also found a trend for a negative association between weight and cognitive change in the A- group. This finding could be related to previous evidence showing that weight loss may have a beneficial effect on cardiovascular health, which in turn can help to preserve cognitive function [50, 51]. However, this hypothetical beneficial effect would be overridden by AD-related cognitive impairment in those who are in the disease *continuum*.

The present study may also help to understand previous findings from epidemiological studies reporting that BMI behaves as a risk factor for dementia when measured during midlife, while this association is reverted in late-life [11, 52–57]. Our findings showing how AD pathology modifies the association between weight and cognitive performance longitudinal changes are consistent with the hypothesis that modifications in the association between BMI and dementia risk across lifespan are driven by a progressive increase in the prevalence of AD pathological change with aging [9]. Importantly, as our study involved mostly middle-aged adults, our results suggest that the confounding effect of AD pathology in the association between BMI and dementia risk is already present in cognitively unimpaired middle-aged population, which is relevant in terms of interpreting previous results and designing future studies in the field, in order to account for the effects of reverse causality.

### Limitations

We must acknowledge several limitations in our study. First, we cannot exclude a selection bias on our results, as they are based on a research cohort mostly composed by middle-aged, well-educated individuals with very low comorbidity burden [23]. Therefore, our results cannot be directly extrapolated to the general population and should be confirmed in independent cohorts. Second, our results were adjusted by different variables related to cardiovascular health and mood, which can influence body weight, but we cannot rule out other causes of unintentional weight loss. Finally, as the present study lacked baseline AD biomarkers data, our findings that weight loss in preclinical AD mainly occurs when both Aβ and tau pathology are present should be confirmed in studies with longer follow-up duration and longitudinal biomarker data. The currently ongoing follow-up visits of ALFA+ study participants will undoubtedly shed light into this.

## Conclusions

In middle-aged cognitively unimpaired adults with a mean follow-up of 4.1 years, weight loss was associated with increased risk of displaying abnormal AD biomarkers levels, as well as faster cognitive decline among those in the Alzheimer’s *continuum*. Weight loss may indicate greater risk of AD-related cognitive impairment among cognitively unimpaired adults at risk for AD and could be useful in order to fine-tune dementia risk scores and for enrichment strategies in studies focused in preclinical AD.

## Data Availability

The data that support the findings of this study may be available on upon reasonable request from the corresponding author.

## Abbreviations

Aβ: Amyloid-β
AD: Alzheimer’s disease
AT: Amyloid, tau
BMI: Body mass index
CSF: Cerebrospinal fluid
FDR: False discovery rate
FTM-PET: [^18^F] flutemetamol PET
HADS: Hospital Anxiety and Depression Scale
MET: Metabolic equivalent of task
NfL: Neurofilament light
OR: Odds ratio
PACC: Preclinical Alzheimer’s Cognitive Composite
PET: Positron emission tomography

## References

[1] McKhann GM, Drachman D, Folstein M, Katzman R, Price D, Stadlan EM (1984). Clinical diagnosis of Alzheimer’s disease: report of the NINCDS-ADRDA Work Group under the auspices of Department of Health and Human Services Task Force on Alzheimer’s Disease. Neurology..

[2] White H, Pieper C, Schmader K, Fillenbaum G (1996). Weight change in Alzheimer’s disease. J Am Geriatr Soc.

[3] White H, Pieper C, Schmader K (1998). The association of weight change in Alzheimer’s disease with severity of disease and mortality: a longitudinal analysis. J Am Geriatr Soc.

[4] Cronin-Stubbs D, Beckett LA, Scherr PA, Field TS, Chown MJ, Pilgrim DM (1997). Weight loss in people with Alzheimer’s disease: a prospective population based analysis. BMJ..

[5] Besser LM, Gill DP, Monsell SE, Brenowitz W, Meranus DH, Kukull W (2014). Body mass index, weight change, and clinical progression in mild cognitive impairment and Alzheimer disease. Alzheimer Dis Assoc Disord.

[6] Barrett-Connor E, Edelstein SL, Corey-Bloom J, Wiederholt WC (1996). Weight loss precedes dementia in community-dwelling older adults. J Am Geriatr Soc.

[7] Stewart R, Masaki K, Xue Q-L, Peila R, Petrovitch H, White LR (2005). A 32-year prospective study of change in body weight and incident dementia: the Honolulu-Asia Aging Study. Arch Neurol.

[8] Buchman AS, Wilson RS, Bienias JL, Shah RC, Evans DA, Bennett DA (2005). Change in body mass index and risk of incident Alzheimer disease. Neurology..

[9] Johnson DK, Wilkins CH, Morris JC (2006). Accelerated weight loss may precede diagnosis in Alzheimer disease. Arch Neurol.

[10] Knopman DS, Edland SD, Cha RH, Petersen RC, Rocca WA (2007). Incident dementia in women is preceded by weight loss by at least a decade. Neurology..

[11] Singh-Manoux A, Dugravot A, Shipley M, Brunner EJ, Elbaz A, Sabia S (2018). Obesity trajectories and risk of dementia: 28 years of follow-up in the Whitehall II Study. Alzheimers Dement.

[12] Alhurani RE, Vassilaki M, Aakre JA, Mielke MM, Kremers WK, Machulda MM (2016). Decline in weight and incident mild cognitive impairment. JAMA Neurol..

[13] Buchman AS, Schneider JA, Wilson RS, Bienias JL, Bennett DA (2006). Body mass index in older persons is associated with Alzheimer disease pathology. Neurology..

[14] Ishii M, Iadecola C (2015). Metabolic and non-cognitive manifestations of Alzheimer’s disease: the hypothalamus as both culprit and target of pathology. Cell Metab.

[15] Jimenez A, Pegueroles J, Carmona-Iragui M, Vilaplana E, Montal V, Alcolea D (2017). Weight loss in the healthy elderly might be a non-cognitive sign of preclinical Alzheimer’s disease. Oncotarget..

[16] Rabin JS, Shirzadi Z, Swardfager W, MacIntosh BJ, Schultz A, Yang H (2020). Amyloid-beta burden predicts prospective decline in body mass index in clinically normal adults. Neurobiol Aging.

[17] Xu W, Sun F-R, Tan C-C, Tan L (2020). Weight loss is a preclinical signal of cerebral amyloid deposition and could predict cognitive impairment in elderly adults. J Alzheimers Dis.

[18] Vidoni ED, Townley RA, Honea RA, Burns JM (2011). Alzheimer disease biomarkers are associated with body mass index. Neurology..

[19] Ewers M, Schmitz S, Hansson O, Walsh C, Fitzpatrick A, Bennett D (2012). Body mass index is associated with biological CSF markers of core brain pathology of Alzheimer’s disease. Neurobiol Aging.

[20] Hsu DC, Mormino EC, Schultz AP, Amariglio RE, Donovan NJ, Rentz DM (2016). Lower late-life body-mass index is associated with higher cortical amyloid burden in clinically normal elderly. J Alzheimers Dis.

[21] Sun Z, Wang Z, Sun F, Shen X, Xu W, Ma Y (2020). Late-life obesity is a protective factor for prodromal Alzheimer’s disease: a longitudinal study. Aging (Albany NY).

[22] Milà-Alomà M, Salvadó G, Gispert JD, Vilor-Tejedor N, Grau-Rivera O, Sala-Vila A (2020). Amyloid beta, tau, synaptic, neurodegeneration, and glial biomarkers in the preclinical stage of the Alzheimer’s continuum. Alzheimers Dement.

[23] Molinuevo JL, Gramunt N, Gispert JD, Fauria K, Esteller M, Minguillon C (2016). The ALFA project: a research platform to identify early pathophysiological features of Alzheimer’s disease. Alzheimer’s Dement (NY).

[24] Papp KV, Rentz DM, Orlovsky I, Sperling RA, Mormino EC (2017). Optimizing the preclinical Alzheimer’s cognitive composite with semantic processing: the PACC5. Alzheimer’s Dement (NY).

[25] Lim YY, Snyder PJ, Pietrzak RH, Ukiqi A, Villemagne VL, Ames D (2016). Sensitivity of composite scores to amyloid burden in preclinical Alzheimer’s disease: introducing the Z-scores of attention, verbal fluency, and episodic memory for nondemented older adults composite score. Alzheimer’s Dement (Amst).

[26] Jonaitis EM, Koscik RL, Clark LR, Ma Y, Betthauser TJ, Berman SE (2019). Measuring longitudinal cognition: individual tests versus composites. Alzheimer’s Dement (Amst).

[27] Donohue MC, Sperling RA, Salmon DP, Rentz DM, Raman R, Thomas RG (2014). The preclinical Alzheimer cognitive composite. JAMA Neurol.

[28] Quintana JM, Padierna A, Esteban C, Arostegui I, Bilbao A, Ruiz I (2003). Evaluation of the psychometric characteristics of the Spanish version of the Hospital Anxiety and Depression Scale. Acta Psychiatr Scand.

[29] Molina L, Sarmiento M, Peñafiel J, Donaire D, Garcia-Aymerich J, Gomez M, et al. Validation of the Regicor Short Physical Activity Questionnaire for the adult population. Lucía A, editor. Plos One. 2017;12:e0168148. 10.1371/journal.pone.0168148 PMC5234797 28085886

[30] Schröder H, Fitó M, Estruch R, Martínez-González MA, Corella D, Salas-Salvadó J (2011). A short screener is valid for assessing Mediterranean diet adherence among older Spanish men and women. J Nutr.

[31] Palmqvist S, Mattsson N, Hansson O (2016). Cerebrospinal fluid analysis detects cerebral amyloid-β accumulation earlier than positron emission tomography. Brain..

[32] Jack CR, Bennett DA, Blennow K, Carrillo MC, Dunn B, Haeberlein SB (2018). NIA-AA research framework: toward a biological definition of Alzheimer’s disease. Alzheimers Dement.

[33] Salvadó G, Molinuevo JL, Brugulat-Serrat A, Falcon C, Grau-Rivera O, Suárez-Calvet M (2019). Centiloid cut-off values for optimal agreement between PET and CSF core AD biomarkers. Alzheimers Res Ther.

[34] Klunk WE, Koeppe RA, Price JC, Benzinger TL, Devous MD, Jagust WJ (2015). The Centiloid Project: standardizing quantitative amyloid plaque estimation by PET. Alzheimer’s Dement.

[35] Bays HE, Chapman RH, Grandy S (2007). The relationship of body mass index to diabetes mellitus, hypertension and dyslipidaemia: comparison of data from two national surveys. Int J Clin Pract.

[36] Varkevisser RDM, van Stralen MM, Kroeze W, Ket JCF, Steenhuis IHM (2019). Determinants of weight loss maintenance: a systematic review. Obes Rev.

[37] Brumpton B, Langhammer A, Romundstad P, Chen Y, Mai X-M (2013). The associations of anxiety and depression symptoms with weight change and incident obesity: the HUNT study. Int J Obes.

[38] Benjamini Y, Hochberg Y (1995). Controlling the false discovery rate: a practical and powerful approach to multiple testing. J R Stat Soc Ser B.

[39] Farrer LA (1997). Effects of age, sex, and ethnicity on the association between apolipoprotein E genotype and Alzheimer disease. A meta-analysis. APOE and Alzheimer Disease Meta Analysis Consortium. JAMA J Am Med Assoc.

[40] Kivipelto M, Ngandu T, Laatikainen T, Winblad B, Soininen H, Tuomilehto J (2006). Risk score for the prediction of dementia risk in 20 years among middle aged people: a longitudinal, population-based study. Lancet Neurol.

[41] Li J, Ogrodnik M, Devine S, Auerbach S, Wolf PA, Au R (2018). Practical risk score for 5-, 10-, and 20-year prediction of dementia in elderly persons: Framingham Heart Study. Alzheimers Dement.

[42] Jessen F, Wolfsgruber S, Wiese B, Bickel H, Mösch E, Kaduszkiewicz H (2014). AD dementia risk in late MCI, in early MCI, and in subjective memory impairment. Alzheimers Dement.

[43] Sánchez-Benavides G, Salvadó G, Arenaza-Urquijo EM, Grau-Rivera O, Suárez-Calvet M, Milà-Alomà M (2020). Quantitative informant- and self-reports of subjective cognitive decline predict amyloid beta PET outcomes in cognitively unimpaired individuals independently of age and APOE ε4. Alzheimer’s Dement (Amst).

[44] Müller S, Preische O, Sohrabi HR, Gräber S, Jucker M, Dietzsch J (2017). Decreased body mass index in the preclinical stage of autosomal dominant Alzheimer’s disease. Sci Rep.

[45] Wilson RS, Arnold SE, Schneider JA, Boyle PA, Buchman AS, Bennett DA (2009). Olfactory impairment in presymptomatic Alzheimer’s disease. Ann N Y Acad Sci.

[46] Albanese E, Taylor C, Siervo M, Stewart R, Prince MJ, Acosta D (2013). Dementia severity and weight loss: a comparison across eight cohorts. The 10/66 study. Alzheimers Dement.

[47] Giudici K, Guyonnet S, Rolland Y, Vellas B, de Souto BP, Nourhashemi F (2019). Body weight variation patterns as predictors of cognitive decline over a 5 year follow-up among community-dwelling elderly (MAPT study). Nutrients..

[48] Jack CR, Wiste HJ, Therneau TM, Weigand SD, Knopman DS, Mielke MM (2019). Associations of amyloid, tau, and neurodegeneration biomarker profiles with rates of memory decline among individuals without dementia. JAMA..

[49] Soldan A, Pettigrew C, Fagan AM, Schindler SE, Moghekar A, Fowler C (2019). ATN profiles among cognitively normal individuals and longitudinal cognitive outcomes. Neurology..

[50] Hamman RF, Wing RR, Edelstein SL, Lachin JM, Bray GA, Delahanty L (2006). Effect of weight loss with lifestyle intervention on risk of diabetes. Diabetes Care.

[51] Ngandu T, Lehtisalo J, Solomon A, Levälahti E, Ahtiluoto S, Antikainen R (2015). A 2 year multidomain intervention of diet, exercise, cognitive training, and vascular risk monitoring versus control to prevent cognitive decline in at-risk elderly people (FINGER): a randomised controlled trial. Lancet..

[52] Whitmer RA, Gunderson EP, Barrett-Connor E, Quesenberry CP, Yaffe K (2005). Obesity in middle age and future risk of dementia: a 27 year longitudinal population based study. BMJ..

[53] Fitzpatrick AL, Kuller LH, Lopez OL, Diehr P, O’Meara ES, Longstreth WT (2009). Midlife and late-life obesity and the risk of dementia. Arch Neurol.

[54] Tolppanen A-M, Ngandu T, Kåreholt I, Laatikainen T, Rusanen M, Soininen H (2013). Midlife and late-life body mass index and late-life dementia: results from a prospective population-based cohort. J Alzheimers Dis.

[55] Kivimäki M, Luukkonen R, Batty GD, Ferrie JE, Pentti J, Nyberg ST (2018). Body mass index and risk of dementia: analysis of individual-level data from 1.3 million individuals. Alzheimers Dement.

[56] Floud S, Simpson RF, Balkwill A, Brown A, Goodill A, Gallacher J (2020). Body mass index, diet, physical inactivity, and the incidence of dementia in 1 million UK women. Neurology..

[57] Qizilbash N, Gregson J, Johnson ME, Pearce N, Douglas I, Wing K (2015). BMI and risk of dementia in two million people over two decades: a retrospective cohort study. Lancet Diabetes Endocrinol.

